# Growing Glia: Cultivating Human Stem Cell Models of Gliogenesis in Health and Disease

**DOI:** 10.3389/fcell.2021.649538

**Published:** 2021-03-25

**Authors:** Samantha N. Lanjewar, Steven A. Sloan

**Affiliations:** Department of Human Genetics, Emory University School of Medicine, Atlanta, GA, United States

**Keywords:** gliogenesis, stem cells, neurodevelopment, neurodevelopmental disorders, astrocyte, microglia, oligodendrocyte

## Abstract

Glia are present in all organisms with a central nervous system but considerably differ in their diversity, functions, and numbers. Coordinated efforts across many model systems have contributed to our understanding of glial-glial and neuron-glial interactions during nervous system development and disease, but human glia exhibit prominent species-specific attributes. Limited access to primary samples at critical developmental timepoints constrains our ability to assess glial contributions in human tissues. This challenge has been addressed throughout the past decade via advancements in human stem cell differentiation protocols that now offer the ability to model human astrocytes, oligodendrocytes, and microglia. Here, we review the use of novel 2D cell culture protocols, 3D organoid models, and bioengineered systems derived from human stem cells to study human glial development and the role of glia in neurodevelopmental disorders.

## Introduction

**FIGURE 1:**
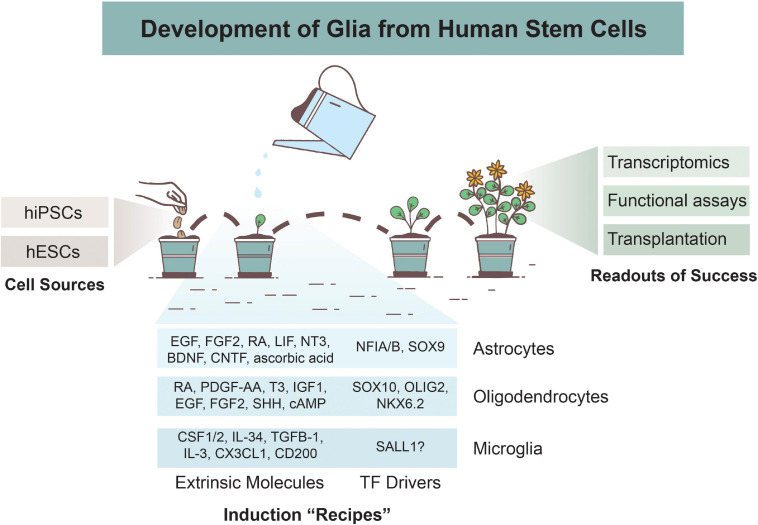
Overview of Glial Development from Human Stem Cells. Astrocytes, oligodendrocytes, and microglia can be derived from human induced pluripotent stem cells (hiPSCs) or human embryonic stem cells (hESCs). Various differentiation protocols have been created to induce glial development via use of extrinsic patterning molecules and/or via induction of transcription factors (TFs). Numerous methods are used to determine successful differentiation and functionality of glial cells, including transcriptomics analyses, functional assays, and xenotransplantations.

**FIGURE 2:**
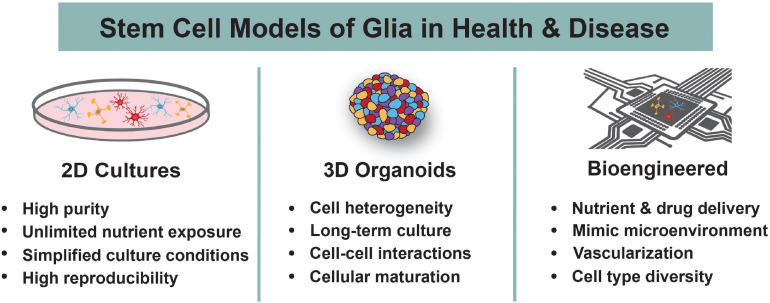
Human Stem Cell Models to Study Glia. Human stem cells are differentiated into astrocytes, oligodendrocytes, and microglia using 2D cultures, 3D organoids, or bioengineered systems. The main advantages of each system are highlighted. These models are used to understand the roles of glia during normal development and in the context of neurodevelopmental disorders.

Glia are essential constituents and regulators of the central nervous system (CNS), and studies from the past several decades have illuminated their immense importance to brain development in both health and disease (Allen and Lyons, 2018). They not only provide trophic, metabolic, and physiological support for neuronal growth and survival but also actively control the development and plasticity of the CNS. Aberrations to the development and function of glia can also have adverse consequences. Recent evidence has implicated each of the major glial cell types, including astrocytes, oligodendrocytes (OLs), and microglia, in the onset and pathogenesis of numerous neurological disorders (Zuchero and Barres, 2015). Although glia have been extensively studied in non-human model systems, the evolutionary divergence of human glia from other species has resulted in stark differences in their functionality, heterogeneity, and molecular characterization (Pinto and Götz, 2007; Thomsen et al., 2016). This translational gap between animal models and humans, in addition to the lack of accessible primary human brain tissue, highlights the need for alternative methods to study human glia. Advancements to *in vitro* stem cell models of the human nervous system have greatly alleviated this problem. Both human embryonic stem cells (hESCs) and human induced pluripotent stem cells (hiPSCs) can be differentiated into neurons and/or glia using various culturing techniques that are amenable to user-defined customizations (Figure 1). Currently, all major glial subtypes can be grown in 2D, 3D, or bioengineered cultures, although to varying levels of purity and efficacy (Figure 2). Throughout this article, the term “glia” will be used in specific reference to astrocytes, OLs, and microglia within the CNS. In this review, we discuss the use of human stem cell-based models to study human glial development and the role of glia in neurodevelopmental disorders (NDDs). We begin by summarizing normal development of human astrocytes, OLs, and microglia. To provide context for the need for human stem cell-based methodologies, we also briefly discuss human-specific attributes of glia. Lastly, we summarize current 2D, 3D, and bioengineered human stem cell-derived models of astrocytes, OLs, and microglia and how these models are used to study the contributions of glia to NDDs.

## Glial Development

There are two primary classes of CNS glia, each with unique developmental origins: macroglia and microglia. Macroglia refer to a class of neural cells within the CNS that share a common neuroectodermal origin with neurons (Reemst et al., 2016). The two most abundant macroglial cells are astrocytes and OLs. Microglia, in contrast, are the resident immune cells of the CNS and are derived from extra-embryonic mesoderm surrounding the yolk sac (Ginhoux et al., 2013). This distinction between macro- and microglia is critical for stem cell-based protocols of glial development, which must replicate these developmental origins during *in vitro* differentiation.

### Astrogenesis and Oligodendrogenesis

During human fetal CNS development, neural stem cells (radial glia) differentiate first into neurons and then astrocytes and OLs in a temporally restricted sequence. Neurogenesis begins early, around 6-8 gestation weeks, in the human fetus (Lenroot and Giedd, 2006). Around 16-18 gestational weeks, radial glia transition to the formation of astrocytes and OLs in a process called gliogenesis. Gliogenesis begins with the production of immature astrocytes, followed by the production of oligodendrocyte precursor cells (OPCs) around 18-20 gestational weeks (Jakovcevski et al., 2009; Zhang Y. et al., 2016). This neurogenic to gliogenic cell fate transition of radial glia is deemed the “gliogenic switch” (Molofsky and Deneen, 2015).

Although it is not fully known what drives the gliogenic switch, a combination of extrinsic, intrinsic, and epigenetic signals have been implicated from studies across multiple model systems. Early rodent studies identified activators of the Janus kinase/signal transducer and activator of transcription (JAK-STAT) pathway, including ciliary neurotrophic factor (CNTF), leukemia inhibitory factor (LIF), and cardiotrophin 1, as cytokines involved in the initiation of astrogenesis (Bonni et al., 1997; Barnabé-Heider et al., 2005). Bone morphogenic protein (BMP) and Notch signaling have also been shown to promote astrogenesis via partial collaboration with JAK-STAT (Nagao et al., 2007). Intrinsically, astrocyte-promoting transcription factors (TFs) are also key regulators of the gliogenic switch. These include proteins like SOX9, NFIA, ATF3, RUNX2, FOXG1, and COUP-TFI and II (Naka et al., 2008; Kang et al., 2012; Tiwari et al., 2018; Falcone et al., 2019), amongst others (Kanski et al., 2014; Takouda et al., 2017). Additionally, chromatin modifications and demethylation of STAT binding sites on astrocyte gene promoters are needed for expression of astrocytic genes like glial fibrillary acidic protein (GFAP) (Takizawa et al., 2001; Namihira et al., 2009). With respect to OLs, extrinsic signals including thyroid hormones, glucocorticoids, and retinoic acid (RA) are all vital to the timing and efficacy of OL differentiation. Additionally, fibroblast growth factor (FGF), sonic hedgehog (SHH), RA, and Notch1 facilitate OPC production and proliferation via induction of TFs like OLIG2 (Barres et al., 1994; Bilican et al., 2008; Ortega et al., 2013). Insulin-like growth factor 1 (IGF1) has similarly been shown to promote oligodendrogenesis and myelination through its receptor signaling cascade (Zeger et al., 2007). These examples are a small subset of the factors that drive the gliogenic switch during development, reviewed extensively in Miller and Gauthier (2007) and Okano and Temple (2009), and are the same signaling molecules and TFs that have been repurposed to drive *in vitro* astrocyte and OL differentiation from human stem cells.

### Microglial Ontogeny

Embryonic yolk sac progenitors are the precursors to all tissue-resident macrophages, including microglia. Prior to the gliogenic switch, microglia have already invaded the CNS, making them one of the first CNS glial residents of the nervous system. Primitive ameboid-like microglia have been observed in the human brain as early as 5 gestational weeks, thus developing side-by-side with neurons prior to the presence of astrocytes and OLs (Verney et al., 2010). Microglial migration and local proliferation continue within the CNS until around 24 gestational weeks.

Environmental cues drive tissue-specific gene expression and endow resident macrophages with specialized attributes. Microglia, as CNS residents, exhibit their own molecular repertoire that is maintained by CNS signals (Bohlen et al., 2017) and are unique to other myeloid cells or monocytes recruited to the CNS (Bennett et al., 2018). For example, *Sall1* has been identified as a microglia-specific signature gene that is not expressed in other mononuclear phagocytes or CNS cells (Buttgereit et al., 2016). Therefore, it important to understand the specific CNS signals that induce microglial phenotypes in order to replicate these attributes *in vitro*. TGF-β signaling is one such pathway that has been characterized as essential for microglial development and the maintenance of microglial-specific markers that distinguishes these cells from other macrophages (Butovsky et al., 2014). Also, multiple studies have shown a strong reliance on macrophage colony-stimulating factor receptor (CSF1-R) for microglial proliferation and survival. CSF1 and interleukin cytokines, such as IL-34, bind to CSF1-R and regulate microglial development (Wang et al., 2012). Mice with null mutations in CSF1 have deficiencies in microglia propagation (Otero et al., 2009), supporting its role as an important regulator of microglial survival, growth, and maturation. Additionally, macrophage migration inhibitory factor and the chemokines CX3CL1 and CXCL12 have been shown to influence microglial recruitment and colonization of specific regions within the CNS (Arnò et al., 2014; Li and Barres, 2018). Moreover, factors secreted by astrocytes, specifically CSF1, IL-34, TGF-β2, and cholesterol, are required *ex vivo* for primary microglia to survive *ex vivo* (Bohlen et al., 2017). Thus, understanding the essential components involved in microglial specification and development greatly aid efforts to use stem cell sources to differentiate microglia in culture and maintain their molecular and phenotypic profiles.

## Human-Specific Features of Glia

Nearly all biological model systems have been used to study glial development and neuron-glial interactions, and this pioneering work has been extensively reviewed in rodents (Zuchero and Barres, 2015), flies (Yildirim et al., 2019), worms (Singhvi and Shaham, 2019), and zebrafish (Lyons and Talbot, 2015). The work summarized in these reviews (and others) has established the foundational framework by which we attempt to understand human glia.

Throughout evolutionary time, increased complexity of the nervous system has been accompanied by more numerous and diverse glial populations (Lago-Baldaia et al., 2020). Evolutionary comparisons of human and rodent glia have repeatedly uncovered uniquely hominid features at molecular (Lui et al., 2014; Berto et al., 2019), functional (Zhang Y. et al., 2016), and morphological levels (Oberheim et al., 2012). For instance, human cortical astrocytes are larger, more structurally complex, more diverse, and able to extend more processes than those of rodents (Oberheim et al., 2009). There are also subtypes of astrocytes that exist exclusively in the hominid brain, such as interlaminar astrocytes (Colombo and Reisin, 2004). Additionally, the human brain has a disproportionately larger volume of white matter compared to other primates (Schoenemann et al., 2005) due to increased levels of myelin within the CNS, which is produced by OLs. This increased heterogeneity may arise, in part, from a unique developmental feature- an abundant population of outer radial glia (oRGs). oRGs are thought to be a major contributor to the expansion of the neocortex in humans because of their increased proliferative potential (Lui et al., 2011). oRGs can give rise to neurons, astrocytes, and OLs (Hansen et al., 2010; Pollen et al., 2015; Huang et al., 2020), although the extent of each remains debated. These various changes observed in the human CNS appear to be autonomous, as engraftment of human astroglial progenitors into rodent brains maintain their human-specific morphologies and even contribute to synaptic and behavioral changes in recipient animals (Han et al., 2013).

As brain complexity and heterogeneity has evolved across species, neurological disorders have also become more complex and subtle in their presentations. Many CNS disorders and diseases are polygenic (McCarroll and Hyman, 2013), meaning multiple genes interact to result in a given phenotype. Even when causal genes are conserved across animal model systems, the genomic landscape (i.e., length, folding, machinery, and epigenetics) of the human genome differs from that of other species (Khoueiry et al., 2017). This leads to uncertainty to whether some genes implicated in neurological disorders in humans behave and interact similarly in non-human model systems.

## Limitations to Studying Human Glia

To properly study genetic interactions and the contributions of glia to human health and disease, we need access to human samples. However, the critical timepoints in which CNS glia develop and proliferate occur during fetal stages of human brain development when access to fetal tissue is restricted or difficult to obtain. In the context of disease studies, postmortem brain tissue is the current gold standard. Postmortem cortex has been used to identify the roles of activated glia in NDDs, such as autism spectrum disorder (ASD) (Edmonson et al., 2014), and neurodegenerative diseases, such as amyotrophic lateral sclerosis (ALS) (Tam et al., 2019). However, due to the age, diseased state, and postmortem intervals from which these samples are collected, they are limited in insight into how glia may initiate pathogenesis and contribute to aberrant CNS physiology. It is also unknown whether the rapid cellular changes that occur upon death affect these analyses and findings. Interestingly, many of the processes involved in development, such as synaptic pruning by microglia and astrocytes, are recapitulated in neurodegenerative pathologies, like Alzheimer’s disease (AD) (Stephan et al., 2012). Thus, more research is needed to study the effects of abnormal glial development on the propagation of both NDDs and neurodegenerative diseases. This makes it even more imperative to have accessible models in which we can study and manipulate glial function and development over relatively long periods of time.

An alternative solution to studying human CNS glia is to utilize stem cells from human patients. Stem cells can be differentiated into the various cell types of the brain, including radial glia, neurons, astrocytes, OLs, and microglia. Recent advancements to stem cell technologies, outlined in detail in the following sections, enable functional modeling of the human brain using an *in vitro* system that recapitulates *in vivo* features. The ability to study glia using human stem cell models is critical to gaining insights into their essential operations within the brain.

In the sections below, we will focus on stem cell models of human glia. We will start by discussing various sources of stem cells and their advantages for disease modeling. Next, we will highlight common metrics used to measure “success” in generating glia *in vitro*. With this pretext, we walk through current 2D, 3D, and bioengineered approaches for generating astrocytes, OLs, and microglia and studying their contributions to NDDs.

## Studying Neurological Disorders Using Human Stem Cell Models

The two primary stem cell varieties for modeling brain-derived cells include hESCs and hiPSCs. Both have the ability to undergo undifferentiated proliferation and can be differentiated into all three embryonic germ layers: ectoderm, mesoderm, and endoderm (Parrotta et al., 2017). Due to the fact that hESCs are derived from human embryos, there is ethical controversy regarding their use. They also require donations from patients undergoing *in vitro* fertilization, so there is a limited number of existing and new hESC lines. Alternatively, hiPSCs are reprogrammed from adult fibroblast cells using some combination of the Yamanaka TFs: Oct3/4, Sox2, c-Myc, and Klf4 (Takahashi and Yamanaka, 2006). They carry the same genomic information as the patient in which they were derived from, resulting in some hiPSC lines exhibiting a propensity to differentiate into certain germ lineages. hiPSCs are also more prone to genetic and epigenetic abnormalities compared the hESCs due to the reprogramming process. Both hESCs and hiPSCs have been used for glial differentiation paradigms (Figure 1), and thus far, no clear evidence suggests that one source is more effective than another in terms of gliogenic potency.

One of the biggest advantages to using human stem cells is the ease in modeling neurological disorders. This is because it is possible to collect fibroblasts, blood, or urine directly from patients with a specific neurological disorder and reprogram them into hiPSCs, which then harbor all of the genetic information of the patient. However, throughout the past decade, recent advancements to gene editing technologies have enabled another increasingly common option– the creation of isogenic lines. Using either control hESCs or hiPSCs, one can use CRISPR/Cas9 to induce specific disease-associated mutations. This approach has the advantage of allowing the user to directly compare control and mutated cells within identical genetic backgrounds. CRISPR and other gene editing tools can similarly be used to study the effects of knocking out genes in control lines or correcting mutations in hiPSCs of diseased patients. These stem cells can then be differentiated into various cell types of the CNS, including neurons and glia, to study neurological disorders in a cell- and disease-specific context. Examples of disease modeling using human stem cell-derived glia are outlined in the sections below and summarized in Table 1.

**TABLE 1:** Using human stem cell models to determine glial contributions to neurodevelopmental disorders.

Disease/Condition	Cell line/Genetic background	Phenotypes	References
Aicardi-Goutières Syndrome (AGS)	hESCs with CRISPR/Cas9-induced frameshift mutations in *TREX1* or hiPSCs from a patient with a homozygous *TREX1* mutation	AGS cortical organoids significantly smaller than controls; AGS astrocytes express elevated levels of neurotoxic type-I interferon genes and cause apoptosis of neurons	Thomas et al., 2017
Alexander’s Disease (AxD)	hiPSCs from AxD patients with *GFAP* mutations	AxD astrocytes inhibit proliferation of control hiPSC-derived OPCs and induce myelination defects	Li L. et al., 2018
Autism Spectrum Disorder (ASD)	hiPSCs or hESCs with doxycycline-inducible shRNAmir-mediated knockdown of *NRXN1*	Impaired astrogenesis following *NRXN1* knockdown in NPCs; neuronal differentiation potential is unchanged	Zeng et al., 2013
	hiPSCs from patients with non-syndromic ASD	ASD astrocytes cause reduced development, morphology, and function of healthy neurons and release increased levels of proinflammatory cytokines and reactive oxygen species	Russo et al., 2018
	hiPSCs from a patient with biallelic deletion of *NRXN1-a*	ASD NPCs proliferate more slowly and preferentially differentiate into astrocytes	Lam et al., 2019a
Down’s Syndrome (DS)	hiPSCs from a DS patient	DS NPCs have decreased neurogenic and increased astrocytic potential when cultured with DS astrocyte conditioned media; DS astrocytes induce neuronal cell death and fail to promote synaptogenesis	Chen et al., 2014
Pelizaeus-Merzbacher Disease (PMD)	hiPSCs from patients with various mutations spanning the *PLP1* gene	Defects in OPC development, OL morphology, and myelination capacity in monolayer cultures and oligocortical spheroids	Nevin et al., 2017; Madhavan et al., 2018
Rett Syndrome (RTT)	hiPSCs from several RTT patients with different *MECP2* mutations	RTT astrocytes reduce the morphology and functionality of wild-type neurons	Williams et al., 2014
	hiPSCs from a patient with a *MECP2* p.Arg294* mutation	RTT astrocytes have reduced acetylated α-tubulin and altered microtubule stability	Delépine et al., 2016
	hiPSCs from female RTT twins with *MECP2* frame-shift mutation	Increased differentiation of astrocytes from RTT NPCs	Andoh-Noda et al., 2015
	hESCs with TALENS-induced *MECP2* loss-of-function mutations	RTT microglia-like cells are significantly smaller in size compared to controls	Muffat et al., 2016
	hiPSCs from male RTT patients with *MECP2* mutations	RTT NPCs have inhibited astrocyte differentiation and decreased neuronal synapse density due to *LIN28* upregulation	Kim J.J. et al., 2019
Schizophrenia (SCZ)	hiPSCs from patients with juvenile-onset SCZ	SCZ hGPCs transplanted into Shiverer mice display deficient myelination, astrogenesis, and astrocyte maturation	Windrem et al., 2017
	hiPSCs from patients with SCZs (including schizoaffective disorder)	Significant reduction of OL production in SCZ lines compared to controls	McPhie et al., 2018
	hiPSCs from patients with *CSPG4* missense mutations	SCZ OPCs display aberrant post-translational processing and subcellular localization of CSPG4/NG2 and reduced morphology, viability, OL maturation, and myelination potential	de Vrij et al., 2019
	hiPSCs from patients with SCZ	SCZ microglia-like cells excessively phagocytose neuronal synapses	Sellgren et al., 2019
	hiPSCs from patients with juvenile-onset SCZ	Defective astrocyte differentiation of SCZ hGPCs due to downregulated BMP signaling	Liu et al., 2019
Tuberous Sclerosis (TSC)	hiPSCs or hESCs with CRISPR/Cas9-induced homozygous or heterozygous mutations in *TSC1* or *TSC2*	Premature and increased astrogenesis in homozygous TSC 2D cultures and cortical organoids; Elevated mTORC1 signaling and STAT3 phosphorylation	Blair et al., 2018

*OPCs, oligodendrocyte precursor cells; NPCs, neural progenitor cells; OL, oligodendrocyte; hGPCs, human glial progenitor cells.*

In addition to disease modeling, human stem cells are commonly used for drug discovery, toxicology screens, and other therapeutically focused assessments. Most drugs are first tested in animal models, with mouse models being the most common. Unfortunately, 90% of new drugs fail during clinical trials due to unforeseen toxicity, efficacy, and safety issues in humans, with therapeutics targeting disorders of the CNS being amongst the highest failure rates (Dowden and Munro, 2019). Supplementing animal model studies with human stem cell experiments could potentially alleviate some of these failures by allowing us to gain a greater understanding of non-neuronal responses to disease and human disease pathogenesis. Although current legislation does not allow for drugs developed *in vitro* to move directly into clinical trials, a transition to adding human stem cell screens in preclinical assessments has thus far been promising (Ross and Wilson, 2014). hESCs and hiPSCs have been comprehensively profiled via genomics, transcriptomics, proteomics, and metabolomics. This work enables opportunities for predictive toxicology and elucidations into toxicity pathways in humans. New drugs can be tested directly in human stem cells or in differentiated CNS cell types to test how drugs specifically affect populations of interest. Additionally, although patients with the same disorder may present with similar phenotypes, certain drugs may only be effective on patients with specific genetic signatures. Personalized medicine in which drugs are tested in patient-specific cells may allow for the identification of which genotypes are therapeutically responsive, allowing for targeted identification of individuals to participate in clinical trials (Han et al., 2011). The use of human stem cells in clinical trials is therefore promising, but further analysis is needed to determine whether *in vitro* systems are accurate enough representations of *in vivo* systems, and if not, what additional features are needed to be adequate for use in preliminary clinical trials.

Another major advantage of human stem cell models is that drug responses can be monitored across a diverse population of patients. It is well known that many disorders affect specific populations and ethnicities at variable frequencies and severities. For example, differences in epilepsy rates in Black (2.13%) vs. White (0.77%) individuals highlights a combination of underlying genetic susceptibilities and socioeconomic access to treatment (Kroner et al., 2013). However, traditional genetics studies are commonly performed in patients from European ancestries and often fail to investigate how disorders disproportionately affect underrepresented groups (Sirugo et al., 2019). The use of human stem cells provides the ability to monitor whether drugs or other therapeutics differentially affect individuals across varying ethnicities.

## Metrics of Success for Validating Glial Identity

One of the major obstacles within the stem cell and glial fields is how we determine “success” of differentiations. Adding to this challenge, there is no current consensus on the appropriate methods or signatures required to define each of the major glial cell types. Here, we will briefly provide perspective on the most common readouts used to measure success of glial differentiation protocols.

The use of antibodies in immunohistochemistry (IHC)/immunocytochemistry (ICC) or with fluorescence-activated cell sorting (FACS) is one of the most frequently used methods to test glial induction. This method is quite simple: detection of a cell type-specific protein within a cell delineates its identity. However, many glial markers lack specificity and/or sensitivity. For example, GFAP is commonly used to detect astrocytes, but the protein is also present in radial glia, is a marker for astrocyte reactivity, and exists in only a subset of differentiated astrocytes *in vivo* (Messing and Brenner, 2020). In the microglial world, TMEM119 has emerged as an excellent microglial-specific marker (Bennett et al., 2016), but *how much* TMEM119 expression is indicative of bona fide microglial identity remains undefined, particularly in humans. Many culture protocols demonstrate induction of TMEM119 protein, but these levels still pale in comparison to *in vivo* quantities. This leads to the question of what thresholds of protein levels are required to prove successful differentiation *in vitro*. In general, these issues emphasize why the use of antibodies as sole markers of glial identity is oftentimes insufficient and further fails to detect cellular heterogeneity. Similarly, glial morphological assessments can be good indicators of cellular maturation and quiescent/healthy vs. reactive/disease states, but they should not be utilized as exclusive readouts. This is because some glial cells share similar morphological features (Zhang, 2001) and because these assessments are highly user dependent.

RNA-sequencing (RNA-seq) is a widely used technique for assessing differentiation success. Transcriptomics analyses allow users to unbiasedly compare expression data of stem cell-derived glia with primary glia (Zhang Y. et al., 2016) and can be used at the single cell level to detect subsets of glial populations. To accompany this transcriptional readout, functional studies are also beneficial to ensure *in vitro* glia acquire the same phenotypes as seen *in vivo*. Functional studies can also be performed via *in vitro* cultures (Cho et al., 2019) and/or xenotransplantation into the mouse brain (Abud et al., 2017). Some may argue that functional studies are the ultimate phenotypic readout of glial identity, although caution should be taken to consider whether the functional comparisons are being made between stem cell-derived glia and *in vitro* models or with *in vivo* observations. This is particularly relevant for cell types (microglia and astrocytes) where *in vitro* phenotypes can diverge from the CNS environment (Foo et al., 2011; Bohlen et al., 2017).

Altogether, combining the use of glial markers, transcriptomic data, and functional studies can help ensure the success of producing glial cells from human stem cells. Metrics of “successful differentiation” remain ambiguous, but it is clear that the combination of each of the above readouts should be a requisite benchmark for claims of successful glial differentiation.

## 2D Glial Stem Cell Models

The use of human stem cells to study glia and their contributions to NDDs has flourished in the last two decades. The generation of glia from human stem cells has unveiled new areas of research into their development, maturation, and involvement in neurological disorders. 2D monolayer culture protocols of glial differentiation from human stem cells remain a steadfast way to obtain astrocytes, OLs, and microglia. These cultures all begin with pluripotent stem cell colonies, sometimes maintained on top of stromal feeders, such as mouse embryonic fibroblasts (MEFs). Stromal feeders support stem cell health but can also result in the transfer of animal pathogens, elicit immune responses, and introduce cell contamination (Llames et al., 2015). As an alternative, more recent protocols have moved toward using feeder-free cultures (Ghasemi-Dehkordi et al., 2015). Feeder-free systems similarly aid in stem cell health through the use of Matrigel-, laminin-, or vitronectin-coated plates. These xeno-free cell culture matrices support the growth and differentiation of human stem cells by mimicking the extracellular environment found in many tissues. In this section, we will highlight current 2D protocols for astrocyte, oligodendrocyte, and microglia differentiation (Table 2) and discuss examples of how these protocols have been used to interrogate glial involvement in NDDs (Table 1).

**TABLE 2:** Recent advancements to 2D and 3D glial differentiation protocols (2015–2020).

Model	Cell source	Key methods	Advantages	Potential limitations	References
**(A)** Astrocytes					
EBs	hiPSCs	Neural induction of neurospheres using 50:50 DMEM/F12 to neurobasal-based media supplemented with AA	Spontaneous production of astrocytes from 3D NPC aggregates in 4 weeks	Relies on spontaneous differentiation into astrocytes, leading to a smaller subset of cells being GFAP^+^ and AQP4^+^; not easily scalable	Zhou et al., 2016
2D	hiPSCs	Culture in ScienCell 1801 Astrocyte Medium with Astrocyte Growth Supplement and 2% FBS	Derivation of astrocyte-like cells from NPCs in 30 days using single medium	Reactivity unknown due to hiPSC-derived astrocytes being compared to A1/A2 murine astrocytes; FBS may induce an active inflammatory state	Tcw et al., 2017
2D	hESCs or hiPSCs	Overexpression of murine *Nfib* and *Sox9* transcription factors	*Nfib* overexpression results in GFAP^+^, VIM^+^, and S100B^+^ astrocytes within 21 days	Unknown if overexpression of human *NFIB* and *SOX9* genes also result in astrocyte production; only qPCR used to validate a subset of astrocyte-specific genes; supplementation with serum can result in reactivity	Canals et al., 2018
2D	hESCs or hiPSCs	Tetracycline-inducible NFIA or NFIA and SOX9 overexpression	Direct conversion of human stem cells into astrocytes in 4-7 weeks; validation via transcriptomics and engraftment methods	Both *GFAP* and S100β expression are induced by NFIA and SOX9, potentially confounding their use as readouts of astrocyte production	Li X. et al., 2018
2D	hESCs or hiPSCs	Transient expression of *NFIA* and addition of macroglial-promoting factors	Transient *NFIA* expression results in macroglial competent cells from NPCs within 5 days	Production of nearly 100% GFAP^+^ cells takes about 75 days total; presence of LIF and FBS can induce *GFAP* expression and astrocyte reactivity	Tchieu et al., 2019
2D	hiPSCs	Culture in N2/B27/insulin media with RA and SAG from days 8-19 then with PDGF-AA, IGF1, T3, and NT3 from days 20-sort; FACS for CD49f^+^ cells	CD49f can be used as a novel reactivity-independent marker to purify astrocytes in 2D and 3D cultures	Possibility that CD49f could also label radial glia populations; use of RA may result in astrocyte reactivity	Barbar et al., 2020
3D	hiPSCs	Neural differentiation of human cortical spheroids and long-term culture (over 1 year)	GFAP^+^, functional, and maturing astrocytes are present after about 7 weeks of differentiation	Endogenous astrogenesis and maturation is slow, indicating a need for faster protocols to derive astrocytes in 3D organoids	Paşca et al., 2015; Sloan et al., 2017
3D	hiPSCs	Culture EBs in induction medium containing KOSR, FBS, and bFGF, followed by cerebral differentiation medium with BDNF added after 1 month	Astrocytes present within 3 months and cultured long-term without hypoxia using optimized neural induction media	Undirected cerebral organoid protocol; culturing with FBS may affect astrocyte reactivity	Quadrato et al., 2017
2D and 3D	hESCs or hiPSCs	3D astrospheres induced via FGF2 and EGF, then matured in single cell monolayers using CNTF; followed by 2D-astrocytes cocultured with 2D-neurons to form 3D organoids	Ability to grow astrocytes in 3D while precisely controlling the numbers and types of cells present	Need to pre-differentiate cells; no endogenous progenitors, so difficult to study the gliogenic switch or influences of neurons on astrocyte production and development	Krencik et al., 2017
**(B)** Oligodendrocytes					
2D	hESCs or hiPSCs	Derive NPCs using BDNF and AA, then differentiate into OLs using PDGF-AA, IGF1, cAMP, T3, FGF8, and Purmorphamine	O4^+^ OPCs can be detected by day 50; BDNF accelerates OL maturation	Culturing on MEFs; only 35% of cells are O4^+^ by day 100; need to FACS sort to enrich for OLs	Piao et al., 2015
2D	hESCs or hiPSCs	Lentiviral induction of NPCs with SOX10, OLIG2, and NKX6.2 transcription factors	Production of 70% O4^+^ OLs from NPCs within 28 days	Must first generate NPCs via EBs; unknown effects of lentiviral presence on OL survival and maturation	Ehrlich et al., 2017
2D	hESCs or hiPSCs	Differentiate into OLIG2^+^ NPCs then transduce with lentiviral SOX10 vector	Induction of SOX10 alone is sufficient to generate functional OLs in 22 days total	Unknown effects of lentivirus and doxycycline presence on OL survival and maturation	García-León et al., 2018
3D	hESCs or hiPSCs	Oligocortical spheroids using PDGF-AA and IGF1 from days 50-60, then addition of T3 days 60-onward	Generate OPCs and myelinating OLs in cortical organoids within 100 days	Due to presence of neurons and astrocytes, myelinating OLs (MYRF^+^) constitute about 20% of cells within the organoids; OLs require over 250 days to mature	Madhavan et al., 2018
3D	hiPSCs	Culture with IWP-2 days 4-24; SAG days 12-24; T3, biotin, NT3, BDNF, HGF, IGF1, PDGF-AA, and cAMP days 25-36; then T3, biotin, cAMP, and AA days 36-onward	Heterogeneous production of OLs within organoids (from pre-OLs to mature, late-stage OLs) by day 160	Requires many patterning molecules; OLs comprise only a subset of total cells at late timepoints	Marton et al., 2019
3D	hESCs or hiPSCs	Inhibit or activate SHH pathway in OLIG2-GFP knock-in human stem cell 3D spheres to obtain dorsal or ventral organoids, respectively	Generation of OLs within brain region-specific (dorsal vs. ventral) forebrain organoids	Requires over 12 weeks to produce OPCs; less than 10% of myelinating cells present by week 18 in fusion organoids; limited studies performed to test functionality	Kim H. et al., 2019
**(C)** Microglia					
EBs and 2D	hESCs or hiPSCs	Induction into primitive microglia via serum-free neuroglial differentiation media with IL-34 and CSF1	Production of MGLs within 75 days with high purity (97%)	Requires positive selection of cells from EB layers and transfer to monolayer culturing for cellular maturation; need for stromal feeders	Muffat et al., 2016
EBs and 2D	hiPSCs	EBs patterned into macrophages with VEGF, BMP4, and SCF, followed by differentiation into MGLs via IL-34, CSF1, and CSF2 microglia medium	MGL production occurs within 45 days with very high yields (10-43x) and purity (100%); no feeder cells required	Requires collecting single cells of embryonic macrophages from yolk sac EB supernatant and culturing in monolayers with or without neurons for maturation into MGLs	Haenseler et al., 2017
2D	hESCs or hiPSCs	Isolation of CXCR1^+^/CD14^+^ MGL progenitors, followed by culture with CSF2 and IL-34	Derivation of microglial progenitors within 45-65 days	Relies on FACS or MACS sorting; lower purity (68%) and yield (2x); lack of microglial maturation	Douvaras et al., 2017
2D	hiPSCs	Differentiation into MGL precursors using CSF1 and IL-3 in low (5%) oxygen for 8-15 days then co-culture with astrocytes or neurons	Co-culture with astrocytes allows for production of functional MGLs within 30-45 days without the need for feeder layers	Lower purity and yield due to co-culturing; relies on other cells for differentiation and maturation rather than a defined set of factors; hypoxia may result in activated microglia	Pandya et al., 2017; Takata et al., 2017
2D or 3D	hiPSCs	Differentiation of HPCs into 2D MGLs using CSF1, IL-34, and TGFβ-1 in low (5%) oxygen for 4 days; co-culture with 3D organoids	High yield of MGLs (30-40/hiPSC) in 35 days without cell contamination (97% purity) in feeder-free conditions	Hypoxic conditions can cause microglia reactivity; FACS step required; MGLs are incorporated into organoids post-differentiation, limiting 3D studies on their development	Abud et al., 2017
2D	hiPSCs	Differentiation of CD43^+^ HPCs using CSF, IL-34, and TGFβ-1, followed by the addition of CD200 and CX3CL1 starting at day 25	Simplified protocol to obtain MGLs within 38 days; avoids co-cultures, hypoxic conditions, complex medias, and FACS	Unable to maintain long-term culture; Must collect and culture floating HPCs; MGLs predominantly do not grow adherently, potentially making downstream studies more difficult	McQuade et al., 2018
3D	hiPSCs	Culture EBs with bFGF for 4 days, transfer to neural induction media, then culture with organoid differentiation media without RA	Innate growth of microglia, macroglia, and neurons within cerebral organoids via minimal patterning	Low yield and variable distribution of microglia throughout the organoids; need additional tests to confirm the functionality and reproducibility of these microglia	Ormel et al., 2018
2D and 3D	hiPSCs	2D differentiation of MGLs using IL-3, CSF2, and FBS, followed by co-culture with dorsal or ventral organoids	Ability to study microglia in brain region-specific organoids	Microglial differentiation protocol is similar to those previously established; potential microglial activation due to culturing with serum	Song et al., 2019

*EBs, embryoid bodies; NPC, neural progenitor cell; MGL, microglia-like cell; HPC, hematopoietic precursor cell; OLs, oligodendrocytes; OPCs, oligodendrocyte precursor cells; KOSR, knockout serum replacement; AA, ascorbic acid; RA, retinoic acid; SAG, smoothened agonist; MEFs, mouse embryonic fibroblasts; HGF, hepatocyte growth factor.*

### Astrocytes

Following early methods to successfully differentiate murine ESCs into cells of the neuronal lineage (Tropepe et al., 2001), similar approaches were sought to generate astrocytes. Many of these astrocyte differentiation protocols from human stem cells are outlined in Table 3 of Chandrasekaran et al. (2016), but here we summarize the general logic amongst popular protocols with a particular focus on those that are new within the past 5 years. The most commonly used neural induction protocol is dual SMAD inhibition in a neurobasal- or DMEM/F12-based media (Chambers et al., 2009; Zhou et al., 2016). This includes either LDN-193189, Noggin, or Dorsomorphin to inhibit the BMP pathway and SB-431542 to inhibit the TGF-β pathway. This dual inhibition helps direct stem cells to a “default” neuroectodermal lineage and to rapidly produce uniform populations of neural progenitor cells (NPCs). In 2D cultures, NPCs organize themselves into structures called neural rosettes, mimicking neural tube-like structures in a dish. While some methods continue directly toward astrocyte differentiation, most protocols require selection and culturing of neural rosettes for a number of passages prior to the initiation of astrogenesis. Thus, the most common approach is to coax astrogenesis from the NPC stage. Almost all current methods use FGF2 (Tabar et al., 2005; Roybon et al., 2013; Li X. et al., 2018) during early expansion stages of NPCs. Epithelial growth factor (EGF) is frequently supplemented as well, although not all protocols add this second growth factor (Song and Ghosh, 2004; Juopperi et al., 2012). At this juncture, 2D astrocyte protocols diverge in terms of which extrinsic gliogenic factors are supplemented into the media. Some protocols utilize serum (Tcw et al., 2017), ascorbic acid (vitamin C) (Palm et al., 2015), RA (Asano et al., 2009), and/or LIF (Tchieu et al., 2019). These additional factors help increase the abundance of GFAP^+^ cells but may simultaneously induce aspects of astrocyte reactivity (Sofroniew, 2020). Once directed toward an astroglial cell fate, some protocols allow time to mature astrocyte progenitors (Lam et al., 2019b) while others direct astrocyte maturation with additional exogenous molecules (Krencik and Zhang, 2011). The former likely produces more bona fide astrocyte-like cells but at the consequence of longer protocols (up to 180^+^ days). To obtain aquaporin-4 (AQP4)^+^ and S100β^+^ cells, markers associated with more mature astrocytes, CNTF and/or brain-derived neurotrophic factor (BDNF) are frequently added to the culture media (Roybon et al., 2013). Many of these supplements are used to differentiate astrocytes because they have been identified as gliogenic inducers in rodents (Johe et al., 1996; Bonni et al., 1997). A caveat of this approach is that most of these factors directly induce *GFAP* expression, making it critical to use orthogonal readouts like functionality and transcriptomic/proteomic profiles to validate the identity of human-stem cell derived astrocytes. Recently, a novel marker of human astrocytes, CD49f, has been identified to help enrich hiPSC-derived astrocytes (Barbar et al., 2020). CD49f can be used to purify primary and *in vitro* astrocytes and serves as a reactivity-independent marker. The identification of additional astrocytic markers will greatly expand our ability to study astrocyte development and to perform higher resolution lineage assessments on astrocyte differentiation.

The protracted process to differentiate human stem cells into functional astrocytes takes around 3 months, and many groups are actively seeking methods to accelerate this process. Tcw et al. (2017) developed a reproducible, single-medium method to generate astrocytes from hiPSCs in less than 30 days (starting with NPCs). It is important to note that they began counting from the NPC stage, while other protocols start counting from the stem cell stage. They tested various media recipes on an astounding 42 hiPSC lines and observed most efficient astrocyte generation using the commercially available ScienCell 1801 medium with Astrocyte Growth Supplement and 1-2% fetal bovine serum (FBS). Although a relatively minor concern, the astrocytes were enriched for some genes associated with reactivity, potentially due to the addition of serum. An additional approach to accelerate the astrocyte differentiation timeline is to force expression of astrocyte-specific TFs like NFIA/B and SOX9 (Deneen et al., 2006; Kang et al., 2012). Several groups have used constitutive or inducible versions of these TFs to “skip” the NPC stage and move directly toward an astrocytic lineage (Caiazzo et al., 2014; Canals et al., 2018; Li X. et al., 2018). Most recently, Tchieu et al. (2019) used transient expression of NFIA in the presence of the gliogenic factor LIF to rapidly induce an astrocyte fate. These NFIA-induced astrocytes exhibit similar morphology, function, chromatin landscape, gene expression profiles, and protein markers to those derived from primary human fetal tissue. Thus, the acceleration of stem cell-derived astrocytes is promising. Deriving astrocytes from NPCs in less than a month compared to 3^+^ months opens the door for more intensive research into their development, maturation, and roles in neurological disorders in a time-saving and cost-effective manner. However, it is important to remain cautious and perform critical analyses to ensure future protocols generate quiescent, non-reactive astrocytes that mimic the growth, development, and functionality of the *in vivo* human brain. Most importantly, astrocyte maturation remains a largely elusive phenotype to achieve in 2D culture. This is a result of the fact that immature astrocytes are still proliferating and dividing, thus requiring continual passaging of monolayer cultures. To alleviate this requirement, we must develop 2D differentiation methods that advance cells beyond this proliferative stage into a mature post-mitotic state.

Many studies using human stem cell-based models of NDDs have implicated critical roles of astrocytes in disease pathogenesis. Deletions in *NRXN1* have been associated with ASD, schizophrenia, and developmental delay. During early stages of neurogenesis, NPCs derived from patient iPSCs with biallelic deletions of *NRXN1-a* have increased astrocytic differentiation potential and impaired neuronal functionality (Lam et al., 2019a). In late stages of neurogenesis, shRNA-mediated knockdown of *NRXN1* in hiPSCs or hESCs leads to impaired astrocyte differentiation, while neuronal differentiation remains largely unchanged (Zeng et al., 2013). Alexander disease, which results from mutations in GFAP, is a primary disorder of astrocytes that leads to neurodevelopmental delays and intellectual disability. Modeling Alexander disease using patient iPSC-derived astrocytes has uncovered that GFAP mutations in astrocytes leads to secondary impaired function and proliferation of OPCs (Li L. et al., 2018). These non-cell autonomous consequences of astrocyte dysfunction have been observed in other disorders as well.

Rett syndrome NPCs derived from female patient iPSCs have increased astrocytic differentiation potential and lower expression of neuronal genes (Andoh-Noda et al., 2015). Contrastingly, Rett syndrome NPCs derived from male patient iPSCs exhibit perturbed astrocyte differentiation and decreased neuronal synapse density (Kim J.J. et al., 2019). Additional studies reveal Rett syndrome hiPSC-astrocytes display decreased levels of acetylated-tubulin (Delépine et al., 2016), potentially leading to unstable microtubules, and have negative effects on the morphology and function of wild-type neurons (Williams et al., 2014). Similarly, when co-cultured with astrocytes generated from non-syndromic ASD patients, otherwise healthy neurons displayed reduced morphology and improper development (Russo et al., 2018). Compared to controls, ASD astrocytes exhibit higher levels of pro-inflammatory cytokines. Using this hiPSC-based model of ASD, the authors identified IL-6 as a potential instigator of neural defects and suggested blockage of the IL-6 pathway as a potential therapeutic approach for a subset of ASD patients. These human stem cell models of NDDs have proven fruitful in revealing how astrocytic dysfunction can influence other cells in the CNS and drive disease pathogenesis. This approach can also expose unknown roles of astrocytes in NDDs. For example, using hiPSCs derived from Down’s syndrome (DS) patients, Chen and colleagues revealed that DS astrocytes exhibit neurotoxic qualities and limited synaptogenic capabilities (Chen et al., 2014). These phenotypes were also partially rescued by a clinically available drug, minocycline. Together, these studies not only uncover how dysfunctional astrocytes disrupt normal structure and function of the CNS, but they also reveal how hiPSC-derived astrocytes and neurons can be used for drug and therapeutics testing.

### Oligodendrocytes

Almost all early protocols of OL differentiation from human stem cells relied on culturing with or collecting culture media from stromal feeder layers like mouse fibroblast cells. Nistor and colleagues published the first protocol to differentiate OLs from hESCs (Nistor et al., 2005). hESCs were grown on Matrigel in a novel glial differentiation media containing IGF1 and triiodothyroidin (T3) and supplemented with EGF, FGF2, and RA to obtain OPCs. This protocol required selection of neurospheres and takes 42 days. After confirming their *in vitro* identity by immunostaining, OLs were transplanted into a Shiverer mouse model of dysmyelination (MBP-/-) to further confirm their functionality *in vivo*. Subsequent studies tested additional supplements, including SHH, Noggin, and/or PDGF-AA, to also successfully obtain OPCs and mature OLs from hESCs (Izrael et al., 2007; Hu et al., 2009). The first detailed OL differentiation protocol from hiPSCs (Wang et al., 2013) used a combination of FGF2, RA, and purmorphamine (SHH agonist) to obtain pre-OPCs, then matured them into OPCs using a glial induction media containing T3, NT3, IGF, purmorphamine, and PDGF-AA, requiring a total of 110-150 days. These hiPSC-derived OPCs have the ability to produce both functional astrocytes and myelinating OLs. Since OLs are derived from NPCs, other groups have pursued protocols that began with neural induction strategies using the dual SMAD inhibition methodology previously described. This approach may provide a more efficient method to obtain pre-OPCs (70-75 days). These methods use similar patterning molecules as previously described, including FGF, PDGF-AA, IGF1, and T3, but additionally incorporate ascorbic acid and cAMP with either RA and SHH (Douvaras et al., 2014) or BDNF (Piao et al., 2015) for OL maturation.

Due to cross-species contamination, high variability, and undefined culture systems, some groups moved away from MEF feeder layers. The first reports of a xeno-free OL culture protocol from hESCs or hiPSCs was published by Sundberg and colleagues (Sundberg et al., 2010, 2011). Their differentiation protocols use IGF1, PDGF-AA, SHH, EGF, FGF2, and CNTF to obtain OPCs in 77-91 days. However, these cultures still rely on human foreskin fibroblast (HFF) feeder cells, and FACS is needed to select for NG2^+^ cells. Ehrlich and colleagues pursued an accelerated method to produce OLs via the use of forced TF expression (Ehrlich et al., 2017). They cultured hiPSCs on MEFs and differentiated them into NPCs using dual SMAD inhibition. The NPCs were then transduced with lentiviral plasmids containing the TFs SOX10, OLIG2, and NKX6.2. Transduced cells were cultured in media supplemented with PDGF-AA, NT3, IGF1, ascorbic acid, and T3. Within 28 days, 70% of the cells were O4^+^. The induced OLs had similar functionality, transcriptomic profiles, and ICC markers as primary human adult OLs. A year later, García-León et al. showed that hiPSC induction of SOX10 alone was sufficient to generate OLs (García-León et al., 2018). They demonstrated a yield of 50-60% O4^+^ cells and the entire process only took 22 days. These O4^+^ cells had similar gene expression profiles to primary human OLs and were able to myelinate neurons both *in vitro* and *in vivo*. Altogether, TF-based methods rapidly accelerate OL differentiation from human stem cells compared to other protocols.

The derivation of OLs from human stem cells in a rapid and efficient manner opens the door for research into disease modeling, drug testing, and therapeutic OL transplantation. There are multiple human CNS disorders that result from aberrant or damaged myelination, and a major therapeutic goal in each of these disorders is remyelination. After establishing TF-mediated differentiation protocols, Ehrlich et al. and García-León et al. tested various drugs with the goal of identifying promyelinating compounds. Using their hiPSC-derived OLs, the authors tested drugs previously identified to promote OL differentiation or myelination in rodents. They validated that some compounds, including clobetasol and miconazole, worked well in a human system, whereas others, such as clemastine, had little to no effect (Ehrlich et al., 2017). Some of these same drugs were also tested in a neuron-OL co-culture system, confirming Ehrlich’s results. Furthermore, using the co-culture system, additional drugs, like pranlukast, have been identified as promising remyelinating compounds for human use (García-León et al., 2018). The ability to test these candidates in an *in vitro* human system allows us to study OL development and maturation and the mechanisms underlying OL-mediated neuronal dysfunction.

The role of OLs in the primary pathogenesis of schizophrenia (SCZ) has been long discussed, primarily due to the identification of abnormalities in and differential expression of myelination-related genes in patients with chronic SCZ (Hakak et al., 2001). With recent advancements to differentiation protocols, OLs can now be derived from SCZ patient hiPSC lines (Liu et al., 2019). O4^+^ SCZ-OLs develop at a significantly lower rate compared to controls (McPhie et al., 2018), suggesting low OPC production as a potential contributing factor to the reduction of white matter in patients with SCZ. A similar study using hiPSCs from SCZ patients with *CSPG4* mutations resulted in OLs with abnormal morphology and reduced viability and myelination potential (de Vrij et al., 2019). When cultured on *ex vivo* brain slices from a Shiverer mouse model of dysmyelination, SCZ-derived OPCs exhibited impaired ability to mature into functional myelin basic protein (MBP)^+^ OLs. Likewise, SCZ hiPSC-macroglial progenitor cells implanted into Shiverer mice resulted in reduced myelination, delayed astrogenesis, and behavioral abnormalities (Windrem et al., 2017). Altogether, deriving OLs from human stem cells is an efficient method for modeling NDDs and testing therapeutics in a human system. It also allows for the potential of transplanting human stem cell derived OPCs or OLs into patients with myelin disorders like Multiple sclerosis.

### Microglia

Generating microglia from human stem cells is more challenging due to their mesodermal developmental origin, which means they do not follow the “default” neuroectodermal differentiation pathway that benefits other CNS lineage glia in traditional NPC cultures. Instead, microglia originate from erythromyeloid progenitor cells of the yolk sac that propagate during hematopoiesis. These progenitors further develop into primitive macrophages that invade the developing neural tube where they transition into microglial progenitors (Kierdorf et al., 2013). In the early 2000s, several methods were established to derive hematopoietic stem cells and myeloid cells from human stem cells, but it was not until 2016 that the first protocol to derive microglia-like cells (MGLs) from hiPSCs or hESCs emerged (Muffat et al., 2016). Muffat and colleagues developed a serum-free neuroglial differentiation (NGD) media that allows for co-culturing of neurons, macroglia, and MGLs. The authors resuspended human stem cells grown on MEFs into 3D “yolk sac” clusters in NGD media containing IL-34 and CSF1, which are vital for microglia differentiation and maintenance *in vivo*. After about a month, these embryoid bodies (EBs) were immunoreactive for markers of early yolk sac progenitors. Upon selection and monolayer culturing in supplemented NGD media, the progenitors formed microglia-like precursors, which differentiated into MGLs after an additional month in culture. Comparisons of hiPSC-derived MGLs to fetal human microglia demonstrated evidence of similar, yet less extensive, genetic signatures and marker expression. Additionally, MGLs functionally resembled fetal human microglia in their ability to phagocytose, react to cytokines and chemokines, and respond to cell damage.

The following year, numerous additional protocols were published to derive MGLs (reviewed in Speicher et al., 2019), including another method that also relies on intermediate EB stages (Haenseler et al., 2017). Three additional protocols grew hiPSC colonies in feeder-free conditions with low (5%) oxygen, a common technique used to generate myeloid progenitor cells (Abud et al., 2017; Pandya et al., 2017; Takata et al., 2017). Pandya and colleagues differentiated hiPSCs into hematopoietic progenitors and then co-cultured floating progenitors with astrocytes to obtain MGLs within one month (Pandya et al., 2017). They used Stemcell Stemdiff APEL medium supplemented with SCF, Flt3L, IL-3, IL-6, CSF3, and BMP4, followed by FBS, IL-3, and CSF1 for microglial differentiation. They demonstrated the functionality of hiPSC-microglia by injecting them into the brains of malignant glioma-bearing mice, resulting in increased survival and injury response capabilities. Takata and colleagues similarly relied on a co-culture system (Takata et al., 2017). They cultured yolk sac macrophage-like cells with human stem cell-derived neurons to differentiate them into MGLs. The process takes about 45 days total, relying predominantly on the neurons and CSF1 supplementation to differentiate microglial precursors into MGLs. Abud and colleagues relied on FACS to sort for CD43^+^ hematopoietic progenitors (Abud et al., 2017). They used an MGL media supplemented with CSF1, IL-34, and TGFβ-1, and later, CD200 and CX3CL1 to differentiate MGLs from the CD43^+^ hematopoietic progenitors. This entire process takes around 40 days but does not rely on co-culture systems, resulting in drastically higher yields and purity. Moreover, they xenotransplanted these stem cell derived MGLs into the cortex of immunocompromised mice and demonstrated their ability to survive long-term (over 2 months) and adapt a quiescent microglial phenotype. Another protocol published by Douvaras and colleagues used a similar approach but instead cultured hiPSCs or hESCs in normoxic conditions (Douvaras et al., 2017). On day 25, after differentiating into MGL precursors, cells were sorted by FACS and further cultured for another 20 days in microglia differentiation media containing CSF2 and IL-34. The resulting MGLs resembled primary human microglia via gene expression, cytokine profiles, and functionality in phagocytosis and calcium release in response to stimulation. In the interim, subsequent studies have been attempted to continue to improve upon these protocols. One updated approach avoids hypoxic incubation, complex media formulation, FACS sorting, and co-culturing, offering a simpler system to generate MGLs (McQuade et al., 2018). Nonetheless, these six protocols to derive MGLs from human stem cells are currently the most widely used within the field and all commonly rely on CSF, IL-34, and/or IL-3.

As the stem cell derived microglial field remains relatively young, it is important to highlight that even these new excellent protocols likely have significant room for improvement. This is largely because we still do not fully understand the intrinsic and extrinsic cues that initiate and maintain bona fide microglial signatures and phenotypes *in vivo*. Many of these preliminary hiPSC-derived approaches may, in fact, represent primitive macrophage populations of the correct ontogeny but lack the necessary cues to exhibit complete microglial identity. Those studies that include transplantation into mice and subsequent transcriptomic profiling have demonstrated molecular signatures that most closely align with the *in vivo* profile, potentially because the necessary maintenance signals are present in that environment. Thus, this engraftment approach could currently be considered the gold-standard for determining the fidelity of stem cell-derived MGL.

The recently established differentiation protocols unlock possibilities to study the roles of microglia in NDDs within a human system. Like astrocytes, microglia have been implicated in Rett syndrome pathogenesis. *MECP2* mutant microglia are neurotoxic and damage dendrites and synapses due to excess glutamate release (Maezawa and Jin, 2010). Muffat and colleagues used Rett syndrome patient-derived iPSCs to produce MGLs and found that *MECP2* mutant MGLs were significantly smaller than wild-type microglia (Muffat et al., 2016). Although no further functional analyses were performed, they demonstrated that their microglia differentiation protocol could be used on hiPSCs derived from disease patients. Human stem cell-derived microglia cultures have also been used to investigate schizophrenia. Using an hiPSC-based model, MGLs derived from schizophrenic patients excessively phagocytosed neuronal synapses (Sellgren et al., 2019), which is consistent with the reduced synapse density phenotype observed in patient postmortem cortical tissue (Glantz and Lewis, 2000). The antibiotic minocycline was also found to reduce schizophrenia-associated microglial synapse uptake, serving as a potential therapeutic for delaying or preventing the onset of synaptic pruning in high-risk patients. Due to the relatively recent establishment of microglial differentiation protocols, there are not yet an abundance of publications using human stem cell-derived microglial cultures to investigate their involvement in NDDs. Many groups have instead used patient hiPSC-microglia to study neurodegenerative diseases and other traumatic injuries to the brain (Haenseler and Rajendran, 2019). Nevertheless, as more groups adapt these protocols, we expect to see a rapid rise in the number of findings of the involvement of microglia in NDDs and therapeutics tests using human stem cell models of MGLs. This is particularly relevant for NDDs in which microglial transplants (depleting resident microglia and replacing them with genetically engineered populations) could offer a new class of therapeutic opportunities.

## 3D Glial Stem Cell Models

There are several limitations to 2D monolayer culturing. In particular, they generate limited cell type diversity, cellular maturation, and tissue organization and require frequent passaging. Therefore, to expand their capabilities, hiPSCs can be formed into 3D spheres and differentiated to form structures called organoids. Organoids better mimic tissue cytoarchitecture, create an environment where cell-cell interactions occur endogenously, and can grow for years (Amin and Paşca, 2018), allowing for increased cellular heterogeneity and prolonged maturation of cells compared to 2D cultures. The use of 3D cell models is not altogether new. Aggregates of stem cells, called EBs, were frequently used to establish many of the 2D differentiation protocols described above. However, EBs were traditionally used to derive cells for downstream monolayer stages. Many groups quickly realized that instead of using EBs as intermediate stages, they could use patterning molecules on these 3D cellular aggregates to form organoids of a desired identity. Protocols using organoids have exploded in the last decade, recapitulating many structures and functions of diverse organs.

The use of brain organoids as a model of neurodevelopment (Di Lullo and Kriegstein, 2017) and the most recent methods used to create brain organoids (Sidhaye and Knoblich, 2020) have been extensively reviewed. This includes modeling various brain regions, the use of extracellular matrices, and neurodevelopmental patterning protocols. However, many organoid-related discussions commonly gloss over the presence and abundance of glial populations within the various systems. In the subsections below, we will evaluate organoid protocols that are specifically used to study the development of astrocytes, OLs, and microglia (Table 2) and their roles in NDDs.

### Astrocytes

One of the inherent limitations of 2D astrocyte cultures is their inability to mature, but this can be circumvented using long-term organoid platforms. 3D aggregates are thought to better maintain stem cell identity and allow for more cell-to-cell interactions, aiding in improved astrogenesis. Additionally, the ability to maintain cultures for long periods of time means that one can simply wait for the gliogenic switch to occur endogenously in these systems. Paşca, Sloan, and colleagues characterized functional quiescent astrocytes within hiPSC-derived cortical organoids grown in serum-free media (Paşca et al., 2015). Relying on supplementation with FGF and EGF followed by BDNF and NT3, this publication provided an efficient and reproducible method to generate and characterize astrocytes within organoids without the need for CNTF, LIF, or serum, which are known activators of astrocyte reactivity. Building upon this protocol, Sloan and colleagues later outlined the developmental and maturation trajectory of astrocytes within the 3D system (Sloan et al., 2017). They isolated astrocytes from human cortical organoids and compared their transcriptomes to primary human astrocytes purified from fetal brain tissue and adult cortical resections using both single cell and bulk RNA-seq (Zhang Y. et al., 2016). At the transcriptomic level, these endogenously formed astrocytes closely mirrored primary human astrocytes. Furthermore, they cultured organoids for almost 600 days and found that around day 175, astrocytes from human cortical organoids start to transcriptionally transition from a fetal to a mature state, recapitulating a nearly identical timeline to the maturation of astrocytes observed during *in vivo* development. Importantly, these *in vitro* astrocyte transcriptomes have largely been compared to *in vivo* cells from surgical resections of non-diseased cortical tissue, which may mask local heterogeneity due to the use of bulk RNA-seq. Thus far, single cell sequencing of cortical organoids has revealed largely homogeneous populations (Quadrato et al., 2017; Sloan et al., 2017), which may not fully recapitulate the diversity of astrocytes observed in the human brain (Darmanis et al., 2015; Batiuk et al., 2020; Fan et al., 2020). Therefore, additional studies will need to fully compare astrocyte heterogeneity within 3D systems to what is found in patient samples using single cell or single nuclear platforms. Another important feature of the cortical organoid system, along with other regionally patterned approaches, is the fact that regional astrocyte heterogeneity across different brain regions can be investigated (i.e., pallium vs. midbrain vs. subpallium). These studies are likely to reveal whether human astrocyte heterogeneity is largely a consequence of developmental origins or local contributions.

The Lancaster protocol is one of the first methods to create whole brain cerebral (undirected) organoids from human stem cells (Lancaster and Knoblich, 2014). Like nearly all organoid protocols, this approach can be used to produce astrocytes at timepoints extending beyond the gliogenic switch (about 100 days). This method relies on growing EBs, embedding them into Matrigel droplets, then transferring them to a spinning bioreactor. In the absence of early specific patterning molecules, this undirected approach is capable of generating multiple brain regions and cell types. This could also mean the presence of more heterogeneous astrocyte populations within individual cerebral organoids, although this has yet to be demonstrated. Using a modified version of this protocol, Quadrato and colleagues demonstrated the production of astrocytes within their organoids (Quadrato et al., 2017). Prior to embedding into Matrigel, EBs were grown in neural induction media containing KnockOut Serum and FBS for 2 days. After 1 month in a spinning bioreactor, BDNF was added to assist with maturation. Using these whole brain organoids cultured for up to 13 months, they demonstrated a sequential progression of cell identities, beginning with radial glia, followed by glutamatergic, GABAergic, and dopaminergic neurons, and finally astrocytes. 6-month-old organoids analyzed using single-cell RNA-seq demonstrated the presence of astrocytes that exhibited mature expression profiles, including the expression of *AQP4* and *GFAP*. Cells from 3-month-old organoids also expressed astrocytic markers but lacked evidence of maturation, indicating a similar endogenous maturation program as previously observed (Sloan et al., 2017).

An alternative, but less common, method to study astrocytes is to grow both neurons and astrocytes separately in 2D cultures and then combine them into 3D spheres (Krencik et al., 2017). In this approach, 2D differentiated neurons and astrocytes derived from human stem cells are plated together and allowed to self-assemble in stationary culture or are assembled into uniform sizes using an Aggrewell plate. One of the main advantages to this system is the ability to study the interactions of neurons and astrocytes in a 3D culture without the presence of other cell types and heterogeneous progenitor populations. This controlled method of 3D co-culturing opens opportunities to further study the roles of astrocytes in neural circuit formation and interactions between astrocytes and other glia. It also provides user control of *when* neurons and astrocytes are combined, which enables precision over the timing of initiating neuron-glial interactions.

These reproducible 3D differentiation protocols from human stem cells, combined with validation of astrocyte development and maturation in the organoid system, provide opportunities for studies into aberrant astrocyte development and function in neurological disorders. One example of this includes the use of cerebral organoids to model early onset Aicardi-Goutières syndrome (AGS), a rare genetic encephalopathy that results in severe intellectual disability in infants (Thomas et al., 2017). Organoids were formed from hiPSCs of patients lacking *TREX1* or hESCs with patient-specific *TREX1* mutations induced using CRISPR/Cas9. AGS-organoids were significantly smaller than controls, recapitulating the microcephaly observed in patients with AGS. AGS-organoids also experienced a high degree of neuronal cell death, which was linked to increased neurotoxic type 1 interferon release by TREX1-deficient astrocytes. Additionally, when healthy control organoids and neurons were cultured in conditioned media from AGS-astrocytes, they experienced increased cell death and organoid size reduction. The authors tested Lamivudine (3TC) and Stavudine (d4T), two FDA-approved HIV antiviral drugs, on TREX1-deficient organoids and observed partial rescue of the neurotoxicity, neuronal death, and size reduction phenotypes. Another recent study utilized cortical organoids with CRISPR/Cas9-induced mutations in hiPSCs to model tuberous sclerosis (TSC) (Blair et al., 2018). TSC is a rare disorder that causes overgrowths (tubers) in the brain and other organs and has high rates of co-morbidities with epilepsy, intellectual disability, and ASD. Organoids null for either *TSC1* or *TSC2* exhibited disrupted suppression of mTORC1 signaling, resulting in the premature initiation of the gliogenic switch. This deficiency in neuronal differentiation, at the expense of excessive astrocyte production, was rescued upon treatment with rapamycin, an mTOR inhibitor. This study was one of the first of its kind to use the organoid system to implicate alterations to the timing of the gliogenic switch in the pathogenesis of an NDD, paving the path for additional studies on the role of aberrant astrocyte development in disease pathogenesis.

### Oligodendrocytes

The ability to study the functions of OLs, particularly axonal wrapping and subsequent myelination, in 2D cultures is limited. This has incentivized the use of 3D cultures as a reproducible system to study OL development and function. Initial protocols to produce brain organoids typically lacked OLs, which was surprising given that OLs are derived from the same radial glia population as astrocytes and neurons. When profiling their organoids using single-cell RNA-seq, several groups observed a small number of endogenous OPCs (Birey et al., 2017; Quadrato et al., 2017), citing the earliest reports of OL-lineage production within organoids. The lack of robust endogenous OPC formation was hypothesized to be a result of the lacking trophic signals, missing differentiations cues, and/or insufficient neuronal activity. The easiest of these hypotheses to test was the supplementation of additional exogenous cues. Thus, a year later, a fully defined protocol for the derivation of functional and mature OLs within brain organoids was published (Madhavan et al., 2018). They utilized a slightly modified version of the Paşca and Sloan protocol (Paşca et al., 2015) to generate cortical spheroids. On day 50, the spheroids were supplemented with media containing PDGF-AA and IGF1 for 10 days to expand the OPC population, followed by T3 for another 10 days to induce OL differentiation. Around day 100 (week 14), these oligocortical spheroids generated robust populations of OLs, while typical cortical spheroids at this age were solely comprised of neurons and astrocytes. By week 20, uncompacted myelin could be detected, with evidence of fully compacting and ensheathing myelination of axons present by week 30. The promyelinating compounds clemastine and ketoconazole were tested in the oligocortical spheroids in lieu of T3. Although all conditions ultimately led to a similar production of OLs, ketoconazole-treated spheroids started myelinating 2 months earlier than T3- or clemastine-treated spheroids. This further corroborated the findings seen in 2D human stem cell cultures that clemastine is not as effective in enhancing myelination in humans as it is in rodents (Ehrlich et al., 2017).

Using modifications to their own organoid differentiation protocol, the Paşca lab also produced human oligodendrocyte spheroids (hOLSs) (Marton et al., 2019). In contrast to the Madhavan and Nevin method, hOLSs were patterned at much earlier timepoints. In addition to patterning via dual SMAD inhibition and EGF/FGF2, the Wnt inhibitor IWP-2 was added to the spheroids from days 4-24 and smoothened agonist (SAG) was added from days 12-24. hOLSs were then cultured with T3, biotin, NT3, BDNF, cAMP, hepatocyte growth factor, IGF1, and PDGF-AA from days 25-36, and from then on in T3, biotin, cAMP, and ascorbic acid. By day 50, NKX2-2^+^/OLIG2^+^ double-positive OPCs and PDGFRα^+^ OPCs could be detected within hOLSs, and these cells continued to mature over the next 100 days into functional myelinating OLs. RNA-seq data revealed similar gene expression between hOLS-derived OL lineage cells and primary human OPCs and OLs isolated via immunopanning, establishing a robust and reproducible protocol that can be used to study OL development in a manner that mimics the *in vivo* cell environment. TF approaches have also been used to generate OLs in brain region-specific organoids. Kim H. et al. (2019) used knock-in OLIG2-GFP human stem cell lines to generate both dorsal and ventral organoids (Birey et al., 2017). Interestingly, dorsal- and ventral-derived OLs exhibited distinct expression patterns. When fused together, dorsally derived OLs outcompeted ventrally derived OLs in long-term culture. The fusion of these two forebrain organoids also promoted increased maturation of OLs and resulted in enhanced myelination potential (Kim H. et al., 2019). Although there are now a handful of 3D differentiation protocols that incorporate OL formation, there are many areas for improvement. These current approaches tend to generate a relatively small number of OLs as compared to neuronal or astrocyte counterparts. This suggests that there are still additional, yet undefined, extrinsic and/or intrinsic cues required to generate robust OL formation, differentiation, and survival. Additionally, testing how neuronal activity within developing organoids affects oligodendrogenesis and OL maturation remains an important next step.

Despite an abundance of evidence implicating OLs in numerous NDDs, including schizophrenia (Chew et al., 2013), Alexander disease (Li L. et al., 2018), and neonatal hypoxic injury (Sowmithra et al., 2020), their involvement in these disorders has yet to be extensively modeled in organoids. This may be due to the difficulty of culturing OLs, even in a 3D system. However, Madhavan et al. (2018) were able to model monogenic leukodystrophy Pelizaeus–Merzbacher disease (PMD) in their oligocortical spheroids using hiPSCs from affected patients. PMD is a rare genetic disease that results from defects in myelin production and causes motor delay, hypotonia, and early childhood mortality. Mutations to and deletions of the disease-causing *PLP1* gene caused significant reductions to the number of OLs present within the oligocortical spheroids. While the mechanistic outcomes behind *PLP1* disruptions have yet to be uncovered, this validated approach provides hope toward future disease modeling using OL-containing organoids to explore patient-specific OL pathogenesis.

### Microglia

Studying the roles of microglia and their development within the brain using 3D neuroectodermal cultures is challenging due to their distinct mesodermal lineage. Groups have largely pursued two approaches to tackle this obstacle: (1) add primary or human stem cell-derived microglia to brain organoids to form multilineage structures or (2) minimally pattern organoids that contain progenitor cells from multiple germ layers. Using the first approach, Abud et al. (2017) used their 2D differentiation protocol (described in the section “2D Stem Cell Models Microglia”) to generate MGLs and co-cultured them with cerebral brain organoids (Lancaster and Knoblich, 2014). Within one week, MGLs were distributed throughout the organoids. To determine whether the MGLs could response to neuronal injury, they mechanically wounded organoids with a needle and observed MGLs clustering at the injury site with morphologies reminiscent of an activated state. Using a similar approach, Song and colleagues asked how microglia interact with neurons in specific regions of the brain and thus generated separate populations of dorsal and ventral patterned organoids (Song et al., 2019). They generated MGLs from hiPSC-derived hematopoietic progenitors using FBS, GM-CSF, and IL-3, then labeled and co-cultured them with each of the two populations of cortical organoids. The authors found that MGLs incorporated at a higher rate into dorsal than ventral organoids, however, upon pro-inflammatory stimuli, MGLs in ventral organoids expressed higher levels of TNF-α, an inflammatory cytokine. Together, this approach of producing 2D microglia and incorporating them into pre-formed organoids has produced promising results. At this stage, it does not appear that these MGL-transplants need to occur at a specific developmental stage of the organoid culture, although additional work is needed to elucidate the developmental consequences of MGL engraftment at various organoid timepoints.

The second approach for introducing MGLs into organoids has focused on innately growing microglia within the organoid structure itself. One study identified the presence of a population of mesodermal precursors within cerebral organoids using single-cell RNA-seq (Quadrato et al., 2017), which was likely the result of remnant spontaneous differentiation. Ormel et al. (2018) established one of the first methods to derive functional microglia within intact cerebral organoids. Using a modified version of the Lancaster protocol (Lancaster and Knoblich, 2014), they formed hiPSCs into EBs using a minimal media to allow for differentiation flexibility while still encouraging a general neuroectodermal trajectory. By day 17, progenitor cells from all 3 germ layers were present. Within another week, mesodermal stem cells began to differentiate into MGLs, as confirmed by transcriptomic comparisons to adult human microglia. By day 50, the organoids contained neurons, astrocytes, and MGLs and exhibited features of cortical lamination. Organoid-derived MGLs also had similar morphologies cell surface markers, and phagocytic abilities as primary microglia, but exhibited elevated inflammatory responses. Overall, Ormel, Vieira de Sá, and colleagues demonstrated that MGLs can be grown intrinsically within organoids, though further work is needed to eliminate organoid-to-organoid variability. They also showed that factors commonly used for 2D microglial differentiation (CSF1, IL-34, IL-3, and TGFβ1) are innately expressed within cerebral organoids, potentially secreted by neurons and/or astrocytes. This method provides novel opportunities to study the development and interactions of microglia in the human brain, although additional trituration is necessary to determine ideal protocols to reproducibly balance the generation of MGLs within organoids without encouraging other non-CNS cell types from forming.

Due to the young age and burgeoning field of incorporating microglia into 3D cultures, there are few current studies investigating the roles of microglia in NDDs using human organoids. However, several groups have used these new approaches to model neurodegenerative disorders like AD pathology. In one study, hiPSCs from unaffected parental *APOE3* patients were induced with homozygous *APOE4* alleles using CRISPR/Cas9 and subsequently differentiated into MGLs (Lin et al., 2018). *APOE3*-MGLs (controls) or *APOE4*-MGLs (AD-risk) were embedded into 2-month-old *APP* duplication organoids and cultured for a month. Organoids with *APOE4*-MGLs exhibited increased accumulation of Aβ aggregates, suggesting that the presence of *APOE4* negatively impacts MGL ability to clear extracellular Aβ. This phenotype was reversible when the *APOE4* mutation was converted to *APOE3*. A simultaneously published study also formed brain organoids from hiPSCs derived from familial and sporadic AD patients and used a dual chamber microfluidics device to study their cellular interactions (Park et al., 2018). This device allowed them to visualize the migration of inactivated primary human microglia into AD-organoids that were otherwise devoid of MGLs. Upon entering the AD-organoids, microglia adapted a reactive phenotype and secreted pro-inflammatory signals. This resulted in toxicity to the AD neurons and astrocytes, causing axonal damage and massive cell loss (up to 40% reduction in organoid surface area). Using two disparate methods of organoid tricultures, groups have begun to elucidate the contributions of microglia in AD pathogenesis. Similar approaches must also be performed to study microglia in the context of NDDs and neuropsychiatric disorders.

## Bioengineered Models

Some of the current major challenges to studying glia using human stem cells include the inability to manipulate the *in vitro* microenvironment of 2D and 3D cultures, the lack of vascularization, and limited progenitor diversity (Centeno et al., 2018). Tackling these obstacles using engineering approaches has proven promising thus far (Cadena et al., 2020) and may provide the key to better recapitulate human neuron/glial development in the future. Below we discuss various features of bioengineered systems that have been applied to improve modeling of human glia.

### Extracellular Matrices

To better mimic the *in vivo* tissue environment, human stem cells and differentiated glia are frequently grown in extracellular matrices. Optimizing these matrices and hydrogels has provided an important opportunity to better maintain glial health. They better mimic the microenvironment of the brain and alleviate the need for feeder layers and serum culturing conditions that are not present in the *in vivo* CNS. In one study, primary human fetal astrocyte progenitors grown in a matrix comprised of collagen, hyaluronic acid, and Matrigel formed radial processes and remained in a quiescent state in *in vitro* culture (Placone et al., 2015). These specific factors were chosen due to the abilities of collagen I to aid in structural support, hyaluronic acid to mimic the extracellular matrix of the brain, and Matrigel to provide endothelial cell compatibility. In another study, hiPSC-derived NPCs were grown in a hydrogel comprised of prepolymer methacrylate-modified hyaluronic acid (Zhang Z.N. et al., 2016). The stiffness of this hydrogel is user-controllable via the intensity and duration of ultraviolet-dependent crosslinking. In addition, small molecules can be added to prepolymer cell suspensions, allowing for precise control over patterning. The authors of this study were able to examine cell migration using two-layered hydrogels, one containing tdTomato-expressing astrocytes and the other containing GFP-expressing NPCs. They discovered that NPCs derived from Rett syndrome patients had an impaired ability to migrate toward neurons and astrocytes, highlighting how bioengineered hydrogel constructs can also be used to study glia in disease states. Additionally, 3D hydrogels have been shown to accelerate the development and differentiation of hiPSC-derived NPCs, serving as a potential tool to obtain glial cells more quickly. Along these lines, Wen and colleagues used a natural hydrogel containing integrin ligand modified alginate to form OL-containing 3D neural spheroids (Wen et al., 2019). They encapsulated hiPSC-derived NPCs in alginate hydrogel, which differentiated into neurons, GFAP^+^ astrocytes, and O4^+^ OLs within 90 days. The cells grown within the hydrogel had high rates of cell viability and differentiation potential, offering another methodology to culture and study human glia. This approach could also lead to improved therapeutics testing by embedding drugs into the hydrogels or matrices to test their effects on healthy and diseased glia.

### Blood Brain Barrier (BBB)

The formation and function of the BBB is coordinated by complex interactions between endothelial cells, pericytes, and astrocytes (Lécuyer et al., 2016). Developing *in vitro* BBB models is crucial for investigating the permeability of potential CNS therapeutics and the contribution of BBB/glial dysfunction in the pathology of neurological disorders (Profaci et al., 2020). Recent efforts have primarily focused on reproducing BBB architecture by vascularizing organoids. One method is to implant human brain organoids into adult mouse brains. Implantation results in microglia and blood vessel integration, progressive cellular maturation, and expanded axonal growth within the organoids (Daviaud et al., 2018; Mansour et al., 2018; Pham et al., 2018). Although xenotransplantation of organoids into rodent brains effectively results in vascularization, there is still a need to investigate BBB formation within these engrafted organoids. Thus, studies exclusively comprised of human cells will also be critical to the advancement of *in vitro* human brain modeling. One study co-differentiated hiPSCs or hESCs into neural and endothelial cells using custom endothelial cell media containing FGF and platelet-poor plasma derived serum for 2-6 days (Lippmann et al., 2012). This resulted in brain microvascular endothelial cells that expressed a subset of canonical BBB markers, formed tight junctions, and restricted cellular and molecular transportation. A similar protocol was later established to form BBB-like structures within organoids from human stem cells differentiated into endothelial cells, astrocytes, and pericytes (Bergmann et al., 2018). 3D BBB models better recapitulate the multicellular interactions within the BBB and can be used to understand the structure and maturation of the BBB over long-term cultures. They also offer a tool for drug discovery and therapeutic testing.

Current vascularization approaches within organoids are generally random in architecture and not yet reproducible. An alternative approach to address this challenge is to utilize microfluidics platforms, also called organs-on-a-chip. Polydimethyl-siloxane (PDMS) has been used to create microchannels on a glass coverslip coated with the cell-adhesive molecule poly-D-lysine (Campisi et al., 2018; Ahn et al., 2020). hiPSC-derived endothelial cells, astrocytes, and pericytes are placed into the main chamber, then additional endothelial cells are seeded into the surrounding microfluidics chambers to traverse the microchannels and create vessel connections within a multicellular environment. This method creates a fluidic 3D BBB microvascular network with tight junctions, low permeability, and the presence of extracellular matrix proteins, consistent with features of the *in vivo* BBB.

## Conclusion and Future Directions

Despite great progress in using human stem cells to model the developing brain, there are still several limitations that 2D, 3D, and bio-engineered models have yet to overcome. One primary challenge with these current methods is difficulty in mass scale-up and standardization. There is considerable variability across different hiPSC and hESC lines and many protocols require several months to produce mature, functional glia. Currently, the field lacks consensus on differentiation protocols due to the fact that we still do not fully understand the *in vivo* cues driving development and maturation of each of these cell types. Additionally, more robust markers to confirm output quality are needed.

Although organoids have aided in advancing cellular complexity and long-term maturation studies, the diversity in cell types and ability to produce fully mature cells still does not match the complexity of the human brain (Bhaduri et al., 2020). At present, *in vitro* cell cultures are unable to fully recapitulate complex cell-cell interactions and do not thoroughly represent the adult glial cell heterogeneity observed *in vivo*. Additionally, due to the limited progenitor pool heterogeneity within 2D and 3D cultures and the limited size expansion of organoids, it can be difficult to reproducibly study whole brain interactions. The incomplete and random nature of the intrinsic cytoarchitecture also serves as a restriction to studying neural circuit formation and aberrant circuitry in disease models. Moreover, *in vitro* studies are limited in their ability to study the influence of systemic changes on CNS physiology. As organoid platforms become more sophisticated, many of these limitations may dissipate. Additionally, there is potential to integrate organoid cultures with other systems to interrogate the influences of external factors, such as the gut microbiome, circulating hormones, and environmental exposures, on gliogenesis and maturation.

In the meantime, one of the biggest unmet needs in the field is reliable glial markers. Many current glial markers are co-expressed by multiple cell types, e.g., S100β is expressed in both astrocytes and OPCs (Deloulme et al., 2004), and/or do not distinguish between developing and terminally differentiated populations, e.g., GFAP is expressed at varying levels in radial glia, immature astrocytes, and mature astrocytes (Middeldorp and Hol, 2011). This oftentimes leads to confounding analyses and results. Therefore, we need new markers with high expression levels and specific antibodies to clearly identify each glial cell of interest at distinct stages of development. This would allow us to answer many of the current open questions in the field, particularly regarding the developmental origins of human glia. Are there glial-restricted precursor cells? What drives the gliogenic switch in humans? What cues initiate and modulate microglial phenotypes in the brain?

We are at an exciting stage where our human glial toolset is expanding faster than our answers to fundamental biological questions about glial development and their contribution to NDDs. As we look to the next decade, we expect that a comprehensive understanding of how glia develop, function, and mature will allow us to better determine the roles of human glia in health and disease.

## Author Contributions

SL composed the manuscript. Both authors developed the sections to be included in the review and edited and finalized the manuscript.

## Conflict of Interest

The authors declare that the research was conducted in the absence of any commercial or financial relationships that could be construed as a potential conflict of interest.
